# Impact of Alcohol Dehydrogenase Gene 4 Polymorphisms on Esophageal Squamous Cell Carcinoma Risk in a Chinese Population

**DOI:** 10.1371/journal.pone.0127304

**Published:** 2015-06-03

**Authors:** Xiaoling Xu, Jiwen Wang, Shuang-Mei Zhu, Ming Yang, Yun Fang, An Zhao, Qian Song, Weimin Mao

**Affiliations:** 1 Cancer Research Institute, Hangzhou, Zhejiang Province, China; 2 Thoracic Surgery Department, Zhejiang Cancer Hospital, Hangzhou, Zhejiang Province, China; 3 Key Laboratory of Diagnosis and Treatment Technology on Thoracic Oncology, Zhejiang Province, China; 4 Department of Radio-Chemotherapy Oncology, Lishui People’s Hospital, The Sixth Affiliated Hospital of Wenzhou Medical University, Wenzhou, Zhejiang, China; 5 College of Life Science and Technology, Beijing University of Chemical Technology, Beijing, China; 6 Department of Medical Oncology, Zhejiang Cancer Hospital, 38 Guangji Road, Hangzhou City, China; 7 Department of Clinical Laboratory, Zhejiang Cancer Hospital, 38 Guangji Road, Hangzhou City, China; Duke Cancer Institute, UNITED STATES

## Abstract

**Background:**

Esophageal squamous cell carcinoma (ESCC) is very common in China and is also one of the most common cancers worldwide. The purpose of this study was to examine the associations between genetic variants of various cancer-related genes and the risk of ESCC.

**Methods:**

In this study, we first examined the association between 18 potentially disruptive genetic variants of 17 genes, including alcohol dehydrogenase 4 (*ADH4*) and checkpoint kinase 2 (*CHEK2*), and ESCC risk in a Hangzhou population of 617 patients matched with 534 controls. Among the 18 single nucleotide polymorphisms (SNPs), two were validated in a Jinan population of 540 patients matched with 550 controls.

**Results:**

Sixteen SNPs in 15 genes, including *CHEK2*, did not have significantly different allele frequency distributions between ESCC patients and control subjects. A significantly increased risk of developing ESCC was revealed in subjects with the AA genotype of rs3805322 (*ADH4*) compared with those with the AG or GG genotype by unconditional univariate logistic regression analysis. Using a dominant model, the CC genotype of rs4822983 (*CHEK2*) had a marginally significant protective effect compared to the CT and TT genotypes. The association of ESCC risk with these two SNPs (rs3805322 and rs4822983) was further validated in a Jinan case-control set. Individuals with the *ADH4* rs3805322 AA or AG genotype had ORs of 1.10 (95% CI = 0.81–1.49, *P* < 0.001) or 1.86 (95% CI = 1.33–2.59, *P* = 0.559), respectively, for developing ESCC compared with individuals with the GG genotype. *CHEK2* rs4822983 CC carriers showed a marginally significantly decreased ESCC risk compared with those carrying the CT and TT genotypes in the validation set (95% CI = 0.61–1.01, *P* = 0.064). However, no evidence of interaction existed between the two SNPs and smoking or drinking in the Jinan case-control set.

**Conclusions:**

In conclusion, this current study provides substantial evidence that genetic polymorphisms of rs3805322 in the *ADH4* gene may be associated with an increased risk of developing ESCC in two Chinese Han populations. Future studies to address the biological function of this polymorphism in the development of ESCC are warranted.

## Introduction

Esophageal cancer (EC) is regarded as one of the most common and fatal malignant tumors in the world. More than 90% of esophageal cancers are esophageal squamous cell carcinomas (ESCCs), which is the most common pathologic type in developing nations [1]. ESCC has a relatively high incidence and morbidity in China compared with western countries [2–4]. Accumulating epidemiological evidence indicates that tobacco smoking, substantial alcohol intake, micronutrient deficiency, and dietary carcinogen exposure can greatly increase the risk of developing squamous cell carcinoma [5]. All these factors can induce or enhance DNA damage, which initiates and/or promotes carcinogenesis. DNA repair has been recognized as the most critical mechanism of protection against DNA damage. Various genes are involved in alcohol-associated carcinogenesis and DNA repair. Single nucleotide polymorphisms (SNPs) in genes such as checkpoint kinase 2 (*CHEK2)* [6] and nei endonuclease VIII-like 2 (*NEIL2*) [7], which contribute to inter-individual diversity in DNA repair capacity, may play a significant role in modifying EC risk [8–10].

The occurrence and development of EC is a multi-stage and multi-factor process involving the accumulation and interaction of various environmental factors and genes. Research on EC-related genes has established that alcohol dehydrogenase 4 (*ADH4)* [11, 12], fibroblast growth factor receptor (*FGFR*) [13], thymidylate synthetase (*TYMS*) [14], and cyclin-dependent kinase inhibitor 1A (*CDKN1A*) [15, 16] are directly involved in EC to various degrees. Genetic variants of mucin 1 (*MUC1*) [17] and S100 calcium binding protein A14 (*S100A14*) [18] have been reported to be associated with ESCC. In addition, abnormal expression of ABI family, member 3 binding protein (ABI3BP) [19]; klotho beta (KLB) [20]; long non-coding RNA metastasis-associated lung adenocarcinoma transcript 1 (MALAT1) [19]; and particular microRNAs, including miR-1206 [20] and miR-612 [21], has been observed in cancer cells.

Considering the importance of these genes in ESCC, we conducted a large case-control study to estimate the association between 18 potential functional genetic variants of 17 genes and ESCC risk in a discovery dataset (Hangzhou population) and further validated two SNPs in a validation dataset (Jinan population).

## Materials and Methods

### Study subjects

To estimate the frequencies of the alleles and genotypes of newly identified polymorphisms, 617 southern Han Chinese patients with ESCC and 534 sex- and age-matched (±5 years) control subjects were recruited from Zhejiang Cancer Hospital (Hangzhou, Zhejiang Province, China) between January 2012 and November 2013. Control subjects were individuals who were seeking health care in the same hospital for non-oncologic diseases. For the validation arm, 540 northern Han Chinese patients with ESCC were recruited between June 2009 and April 2012 from Shandong Cancer Hospital (Jinan, Shandong Province, China). These patients presented with histologically confirmed ESCC. Five hundred and fifty cancer-free control subjects, randomly selected from a cancer-screening program for the early detection of cancer performed in Jinan city, were frequency-matched to the cancer cases by age (±5 year), gender, and residential area. A short questionnaire was used to obtain demographic and risk factor information, including smoking and alcohol status. Smokers were classified as individuals who smoked once per day for more than one year. Subjects were also defined as alcohol consumers if they ingested alcohol at least once per week. Written informed consent was obtained from all the patients enrolled in this study. The study was approved by the ethics review board of Zhejiang Province Cancer Hospital.

### SNP selection and genotyping

We selected SNPs based on their functional potential with a minor allele frequency greater than 0.05 in the Asian population and reviewed related literature to identify potential SNPs that could impact EC.

After signing informed consent forms, each subject donated 5 ml of peripheral blood, which was used for genomic DNA extraction. A Blood Genomic DNA Isolation Kit (Axygen Scientific Inc., CA, USA) was used to extract DNA from leukocyte cell pellets according to the manufacturer’s instructions. The DNA purity and concentration were determined by spectrophotometry. All 18 SNPs that were detected at the first stage in the Hangzhou case-control set (discovery set) were also detected using the MassARRAY system (Sequenom Inc., San Diego, California, USA). The genotyping of rs3805322 (*ADH4*) and rs4822983 (*CHEK2*) in the Jinan case-control set was performed using TaqMan assays on an ABI 7900 system (Applied Biosystems, Foster City, CA) according to the manufacturer’s instructions. Primers and probes for the two SNPs were supplied by Applied Biosystems. Real-time quantitative polymerase chain reaction (qPCR) was performed under the following conditions: 50°C for 2 minutes, 95°C for 10 minutes, and 45 cycles of 95°C for 30 seconds and 60°C for 1 minute. A random selection of fifteen percent of the samples was reciprocally tested by different persons, and the reproducibility was 99.5%.

### Statistical Analysis

Differences in gender, age, lifestyle habits and genotype distributions between patients and control subjects were evaluated using Pearson’s Chi-Square (X^2^) test. Multiple correction (Bonferroni correction) was used to validate the significant variables. The dominant and recessive models were used to assess the risk of SNP genotypes in EC. The reference group was the minor homozygous genotype among the controls. The associations between two SNPs and the risk of EC were estimated by computing the odds ratios (ORs) and their 95% confidence intervals (CIs) from both univariate and multivariate logistic regression analyses. The ORs were also adjusted for age, gender and smoking status where appropriate. The gene-environment interaction was evaluated by one-way analysis of variance. All the tests were two-sided, and *P* < 0.05 was considered statistically significant. All the statistical analyses were performed with the SPSS software package (SPSS 18.0 Inc., Chicago, IL, USA).

## Results

The clinical characteristics of the discovery dataset (Hangzhou case-control set) and the validation dataset (Jinan case-control set) are listed in **Table 1**. These two case-control sets were used to detect associations between five *ADH1B-ADH1C-ADH7* cluster SNPs and the risk of ESCC [8]. There was no difference in the sex or age distribution between ESCC patients and healthy controls for both the Hangzhou and Jinan populations. There were more smokers and alcohol drinkers among the ESCC cases in the Jinan population compared with the controls (both *P* < 0.05). Unfortunately, no data were collected on the smoking and drinking habits of the controls in the Hangzhou population. The Hardy-Weinberg equilibrium (HWE) values of these 18 SNPs in the Zhejiang set are presented in Table 2. In addition, the *P* values of the HWE tests for rs3805322 (*ADH4*) and rs4822983 (*CHEK2*) in the controls of the Jinan set were 0.495 and 0.873, respectively.

**Table 1:** Distribution of selected characteristics among ESCC patients and healthy controls.

Variable	Hangzhou case-control population			Jinan case-control population		
	(Discovery population)			(Validation population)		
	Cases	Controls	*P* ^1^	Cases	Controls	*P* ^1^
	No. (%)	No. (%)		No. (%)	No. (%)	
	617	534		540	550	
**Age (years)** ^**2**^			0.711			0.167
** ≤62 (≤56)**	311 (50.4)	275 (51.5)		271 (50.2)	299 (54.4)	
** >62 (>56)**	306 (49.6)	259 (48.5)		269 (49.8)	251 (45.6)	
**Sex**			0.988			0.193
** Male**	534 (86.5)	462 (86.5)		428 (79.3)	453 (82.4)	
** Female**	83 (13.5)	72 (13.5)		112 (53.6)	97 (17.6)	
**Smoking**			NC			<0.001
** Yes**	425 (68.8)	NA		354 (65.5)	285 (51.8)	
** No**	192 (31.2)	NA		186 (34.4)	265 (48.2)	
**Drinking**			NC			0.001
** Yes**	411 (66.6)	NA		300 (55.6)	251 (45.6)	
** No**	206 (33.4)	NA		240 (44.4)	299 (54.4)	

Abbreviations: NA, not available; NC, not calculated; ESCC, esophageal squamous cell carcinoma.

^1^Two-sided χ^2^ test.

^2^The median ages of the cases in the Hangzhou and Jinan populations were 62 and 56 years old, respectively.

**Table 2:** Allele frequency distribution between the ESCC and control populations.

SNP name	Gene locus	Location	Function	Major/major allele	Major/minor allele	Minor/minor allele	No. of Major/major alleles in cases	No. of Major/minor alleles in cases	No. of Minor/minor alleles in cases	No. of Major/major alleles in controls	No. of Major/minor alleles in controls	No. of Minor/minor alleles in controls	X^2^ test (Hardy-Weinberg test for controls)	P value ((Hardy-Weinberg test for controls))	*P* (Fisher’s test)
**rs3805322**	ADH4	100056998	Intron region	AA	AG	GG	236	256	123	125	246	141	0.744	0.689	**0.000**
**rs4822983**	CHEK2	28719078	Intron region	CC	CT	TT	358	226	32	324	166	23	0.087	0.957	0.225
**rs10082466**	MBL2	52766862	3'-UTR	AA	AG	GG	437	161	15	369	147	13	0.621	0.733	0.844
**rs1152620**	MALAT1	65489858	N/A	AA	AG	GG	188	120	303	149	112	267	161.733	<0.001	0.6
**rs11548103**	S100A14	52766862	3'-UTR	GG	AG	AA	49	286	278	58	231	239	0.038	0.981	0.198
**rs11716316**	ABI3BP	100764451	Intron region	AA	AC	CC	188	138	286	150	117	262	152.489	<0.001	0.6
**rs9327870**	ABI3BP	102667853	N/A	TT	TC	CC	186	258	85	218	281	115	2.079	0.353	0.426
**rs12904**	EFNA1	155134221	Near Gene-5	AA	AG	GG	451	152	11	393	125	11	0.082	0.959	0.862
**rs1321311**	CDKN1A	36655123	N/A	GG	GT	TT	425	166	16	364	146	16	0.085	0.958	0.901
**rs17618244**	KLB	39446909	Missense	GG	AG	AA	415	175	23	372	139	18	1.234	0.54	0.634
**rs2073498**	RASSF1A	50332115	Missense	CC	CA	AA	530	81	4	461	64	5	2.614	0.271	0.74
**rs2114358**	miR-1206	128008933	Intron region	TT	TC	CC	314	181	32	339	238	37	0.315	0.854	0.194
**rs2285947**	DNAH11	21544470	Intron region	GG	GA	AA	263	284	66	232	235	58	0.017	0.991	0.869
rs351855*	FGFR	177093242	Missense	TT	CT	CC	402	5	191	334	14	146	430.686	<0.001	**0.035**
**rs4072037**	MUC1	155192276	Cds-synonymous	AA	AG	GG	383	127	15	441	150	17	0.945	0.623	0.982
**rs550894**	miR-612	65444469	ncRNA	GG	GT	TT	342	238	31	282	208	35	0.163	0.922	0.466
**rs699517**	TYMS	673016	3'-UTR	TT	CT	CC	259	287	67	236	240	53	0.499	0.779	0.698
**rs8191664**	NEIL2	11786044	Missense	GG	GT	TT	400	191	22	355	148	25	3.382	0.184	0.431

Abbreviations: ESCC, esophageal squamous cell carcinoma; SNP, single nucleotide polymorphism; OR, odds ratio; CI, confidence interval.

* Because the number of heterozygous alleles was too small, the P values for comparisons of major/major allele vs. major/minor allele vs. minor/minor allele were not sufficiently persuasive. The comparisons of TT+CT versus CC or TT versus CT+CC were not significant. Thus, we did not further evaluate this SNP.

We genotyped 18 selected SNPs in 17 genes for all 617 ESCC patients and 534 control subjects in the discovery arm (Hangzhou). The location and potential effects of these 18 SNPs, some of which cause amino acid substitutions in the proteins, are listed in **Table 2**. The genotype frequencies of rs3805322 (*ADH4*) polymorphisms were significantly different between cases and controls (*P* < 0.001, **Table 2**). Sixteen SNPs in 15 genes, including *CHEK2*, *MBL2*, *MALAT1*, and *ABI3BP*, showed no significantly different allele frequency distributions between ESCC patients and control subjects based on Fisher’s exact test (**Table 2**). Unconditional univariate logistic regression analysis revealed a significantly increased risk of developing ESCC in subjects with the AA genotype for rs3805322 (*ADH4*) compared with those with the AG (OR 1.19, 95% CI 0.89–1.61) or GG (OR 2.16, 95% CI 1.56–3.00) genotypes. Similar results were obtained using dominant or recessive models, which produced respective ORs of 1.95 (95% CI 1.50–2.52, *P* < 0.001) and 1.52 (95% CI 1.15–2.00, *P* = 0.003). Using the dominant model (CC vs. CT+TT), the CC genotype of rs4822983 had a marginally significant protective effect compared to the CT and TT genotypes (OR 0.81, 95% CI 0.64–1.03, *P* = 0.085). Similar results were observed for the two SNPs (rs3805322 and rs4822983) by multivariate logistic regression analysis, which was adjusted for environmental factors such as smoking and drinking (**Table 3**).

**Table 3:** Genotype frequencies of rs3805322 (ADH4) and rs4822983 (CHEK2) among cases and controls and their association with ESCC risk.

Genotype	Hangzhou case-control set				Jinan case-control set				All patients			
	Cases	Controls	OR^1^ (95% CI)	*P* ^1^	Cases	Controls	OR^1^ (95% CI)	*P* ^1^	Cases	Controls	OR^1^ (95% CI)	*P* ^1^
	No. (%)	No. (%)			No. (%)	No. (%)			No. (%)	No. (%)		
***ADH4***	*n* = 615	*n* = 512		<0.001	*n* = 540	*n* = 550		<0.001	*n* = 1155	*n* = 1062		<0.001
**rs3805322**												
**GG**	123 (20.0)	141 (27.5)	1.00 (Reference)		117 (21.7)	150 (27.3)	1.00 (Reference)		240 (20.7)	291 (27.4)	1.00 (Reference)	
**AG**	256 (41.6)	246 (48.0)	2.20 (1.57–3.02)	<0.001	220 (40.7)	261 (47.5)	1.86 (1.33–2.59)	<0.001	476 (41.2)	507 (47.7)	1.13 (0.92–1.40)	0.248
**AA**	236 (38.4)	125 (24.4)	1.18 (0.88–1.59)	0.276	203 (37.6)	139 (25.3)	1.10 (0.81–1.49)	0.559	439 (38.1)	264 (24.8)	2.01 (1.60–2.53)	<0.001
***CHEK2***	*n* = 616	*n* = 513		0.089	*n* = 540	*n* = 550		0.064	n = 1155	n = 1062		0.011
**rs4822983**												
**CC**	358 (58.1)	324 (63.2)	1.00 (Reference)		224 (41.5)	350 (63.6)	1.00 (Reference)		582 (50.3)	674 (63.4)	1.00 (Reference)	
**CT+TT**	258 (41.9)	189 (36.8)	0.81 (0.63–1.03)		316 (58.5)	200 (36.4)	0.79 (0.61–1.01)		574 (49.7)	389 (36.6)	0.80 (0.67–0.95)	

Abbreviations: ESCC, esophageal squamous cell carcinoma; SNP, single nucleotide polymorphism; OR, odds ratio; CI, confidence interval.

^1^ Multivariate logistic regression was used to evaluate the data adjusted for numerous variables, including age, sex, and smoking and drinking status.

The association of ESCC risk with these two SNPs (rs3805322 and rs4822983) was further validated in an independent case-control set (Jinan). Individuals with the *ADH4* rs3805322 AA or AG genotypes had ORs of 1.10 (95% CI = 0.81–1.49, *P* < 0.001) or 1.86 (95% CI = 1.33–2.59, *P* = 0.559), respectively, for developing ESCC compared with those with the GG genotype. *CHEK2* rs4822983 CC carriers showed a marginally significantly decreased ESCC risk compared with those harboring the CT and TT genotypes in the Jinan population (95% CI = 0.61–1.01, *P* = 0.064) (**Table 3**). The results became more significant when the two population sets were combined (**Table 3**).

Because of the key roles of *ADH4* in ethanol metabolism and of *CHEK2* in DNA damage repair, these two SNPs were further examined by stratifying the subjects in the Jinan cohort by smoking status and alcohol drinking history (**Table 4**). Interestingly, we found no significant association between the *ADH4* rs3805322 genotypes and ESCC risk among the subgroups of either smokers or non-smokers (**Table 4**). There was a significant association between *ADH4* rs3805322 genotype and ESCC risk among alcohol drinkers (OR 2.59, 95% CI 1.76–3.84). However, there was no statistically significant association between the *CHEK2* rs4822983 genotype and ESCC risk. In addition, the gene-environment interactions between the two SNPs (*ADH4* rs3805322 and *CHEK2* rs4822983) and smoking or drinking were evaluated using SPSS. No evidence of interaction existed between the two SNPs and smoking or drinking in the Jinan cohort (**Table 4**).

**Table 4:** Risk of ESCC associated with the rs3805322 (ADH4) and rs4822983 (CHEK2) SNPs by smoking status and drinking history in the Jinan set.

Variable	*ADH4* rs3805322				*P*_interaction_ ^2^	*CHEK2* rs4822983				*P*_interaction_ ^2^
	No. of AA	No. of AG+GG	OR^1^ (95% CI)	*P*		No. of CC	No. of CT+TT	OR^1^ (95% CI)	*P*	
**Smoking status**					0.939					0.366
**No**	112/169	74/96	0.86 (0.58–1.27)	0.443		143/197	97/102	0.76 (0.54–1.09)	0.132	
**Yes**	204/181	104/150	0.78 (0.57–1.08)	0.131		173/153	127/98	0.87 (0.62–1.23)	0.434	
**Drinking status**					0.085					0.302
**No**	87/90	153/209	1.32 (0.92–1.90)	0.131		112/169	74/96	0.86 (0.58–1.27)	0.443	
**Yes**	116/49	184/202	2.59 (1.76–3.84)	<0.001		204/181	150/104	0.78 (0.57–1.08)	0.131	

Abbreviations: ESCC, esophageal squamous cell carcinoma; SNP, single nucleotide polymorphism; No., Number; OR, odds ratio; CI, confidence interval.

^1^ Logistic regression was used to evaluate the data adjusted for numerous variables, including age, sex, and smoking and drinking status.

^2^ The multiplicative interaction term was used to calculate *P* values for gene-environment interactions

## Discussion

Alcohol drinking is one of the most important modifiable lifestyle factors affecting EC, and alcohol metabolism has been suggested to play a central role in esophageal carcinogenesis [19]. Alcohol dehydrogenase enzymes (ADHs), which oxidize alcohol to acetaldehyde, are the most important and representative alcohol-metabolizing enzymes [20].

*ADH4* is a key member of the ADH family of proteins encoded by seven ADH genes, *ADH1A*, *ADH1B*, *ADH1C*, *ADH5*, *ADH4*, *ADH6*, and *ADH7* [21]. *ADH4* exhibits the highest catalytic efficiency in the human ADH family; it may account for as much as 40% of the total ethanol oxidation rate at intoxicating levels of alcohol. The association between EC and polymorphisms in some of these *ADH* genes, including rs1229984 (*ADH1B*), rs698 (*ADH1C*), rs17028973 (*ADH7*), and rs671 (*ALDH2*), has been investigated [8, 22–26].

In our study, we examined the association between 18 selected SNPs in 17 cancer-related genes and the risk of developing ESCC in a two Chinese populations. We found that patients with the AA genotype of rs3805322 (*ADH4*) had a significantly increased risk of developing ESCC compared with those with the AG or GG genotypes in our discovery dataset. Then, we successfully validated this result in a validation dataset (Jinan). In a matched case-control study including 585 patients with upper aerodigestive tract cancer and 1,170 non-cancer outpatients, Oze *et al*.[11] determined that compared with other genotypes, the GG genotype of *ADH4* rs3805322 was associated with an increased risk of upper aerodigestive tract cancer in per-allele, dominant, and recessive models. However, this previous study, which was conducted in a Japanese population, only included 265 EC patients. The results of our study, which included a total of 1155 ESCC patients and 1062 controls, are inconsistent with this report [11].

Among DNA repair genes, *CHEK2* (also known as Chk2 or Cds1) is a checkpoint kinase and transducer of cellular responses to DNA damage [27, 28]. Increasing evidence suggests that *CHEK2* plays an important role in DNA damage signaling networks. In the current study, we observed that the CC genotype of *CHEK2* rs4822983 showed a marginally significantly decreased ESCC risk compared with the CT and TT genotypes. When we combined the two groups, the significance increased. The results of our study are consistent with those of a genome-wide association study (GWAS) conducted by Wu *et al*. [29]. However, there was no interaction between the two polymorphisms (*ADH4* rs3805322 and *CHEK2* rs4822983) and drinking and smoking in terms of ESCC susceptibility.

Potential limitations of this study should be considered. First, this was a moderately sized case-control study with a total of more than 1,000 cases. The statistical power may be limited because of the sample size. Thus, it is important that the observed associations are validated in a larger study. Second, this was a hospital-based study; therefore, selection bias may be unavoidable. Third, data on smoking and drinking status were unknown in the Hangzhou case-control set and were therefore not adjusted for in the logistic regression models. A population-based study is needed to further validate our findings.

## Conclusions

The current study provides substantial evidence that genetic polymorphisms of rs3805322 in the *ADH4* gene may be associated with an increased risk of developing ESCC in two Chinese Han populations. Polymorphisms in *ADH4* rs3805322 influence susceptibility to ESCC in different genetic models of allele-dose effects and recessive effects. Future studies to address the biological function of these polymorphisms in the development of ESCC are warranted.
